# Optimization of lead adsorption of mordenite by response surface methodology: characterization and modification

**DOI:** 10.1186/2052-336X-12-5

**Published:** 2014-01-06

**Authors:** Havva Turkyilmaz, Tolga Kartal, Sibel Yigitarslan Yildiz

**Affiliations:** 1 Environmental Engineering Department, Suleyman Demirel University, Cunur, Isparta, Turkey; 2 Chemical Engineering Department, Suleyman Demirel University, Cunur, Isparta, Turkey

**Keywords:** Risk, Environment, Hazard, Lead, Adsorption

## Abstract

**Background:**

In order to remove heavy metals, water treatment by adsorption of zeolite is gaining momentum due to low cost and good performance. In this research, the natural mordenite was used as an adsorbent to remove lead ions in an aqueous solution.

**Methods:**

The effects of adsorption temperature, time and initial concentration of lead on the adsorption yield were investigated. Response surface methodology based on Box-Behnken design was applied for optimization. Adsorption data were analyzed by isotherm models. The process was investigated by batch experiments; kinetic and thermodynamic studies were carried out. Adsorption yields of natural and hexadecyltrimethylammonium-bromide-modified mordenite were compared.

**Results:**

The optimum conditions of maximum adsorption (nearly 84 percent) were found as follows: adsorption time of 85-90 min, adsorption temperature of 50°C, and initial lead concentration of 10 mg/L. At the same optimum conditions, modification of mordenite produced 97 percent adsorption yield. The most appropriate isotherm for the process was the Freundlich. Adsorption rate was found as 4.4. Thermodynamic calculations showed that the adsorption was a spontaneous and an exothermic process.

**Conclusions:**

Quadratic model and reduced cubic model were developed to correlate the variables with the adsorption yield of mordenite. From the analysis of variance, the most influential factor was identified as initial lead concentration. At the optimum conditions modification increased the adsorption yield up to nearly 100 percent. Mordenite was found an applicable adsorbent for lead ions especially in dilute solutions and may also be applicable in more concentrated ones with lower yields.

## Background

Heavy metals are defined as those having higher density than 5000 g/L [1]. When their concentrations reach a certain level, they cause serious damage to the environment, animals and public health. Lead is one such extremely toxic element even at low concentrations that can damage to the nervous system, gastrointestinal tract, reproductive system, liver and brain [2,3]. Major industries of lead pollution are mining, paint, chemical, textile etc. [4]. Lead discharge from them to the atmosphere is approximately 2000 kilotons/year; thus lead pollution is serious environmental problems of worldwide. There are conventional methods for removing lead from aqueous solution such as reverse osmosis, ion exchange, electrochemical treatment, solvent extraction, chemical precipitation, adsorption and biosorption [5,6]. Among those, adsorption is a simple process with low cost and good performance [7]. High exchange capacity, high specific surface area and low cost make natural zeolites good adsorbents [8,9]. Their adsorption capacity can also be further increased by modification with various agents, such as CTAB (cetyltrimethylammoniumbromide) and HDTMA (hexadecyltrimethylammonium-bromide) [9]. A special type of zeolite, mordenite is abundant in nature. Comparing to other zeolites, it has rather low Si/Al ratio (5:1) which may render the adsorption of lead [10]. Removal of heavy metal ions by using mordenite is very rare [11-14]. None of the studies investigated the effect of modification of mordenite on adsorption yield. Above studies have shown that lead removal by mordenite is strongly dependent upon the initial concentration of lead and adsorption conditions. In assessing the effect (single effect and interactive effects) of variables on quality attributes an adequate experimental design is required. Response Surface Methodology (RSM) has an important application for analyzing effects of several independent variables and also interactive effects among the variables on the response. Nevertheless, no study has been found in the literature for optimization of adsorption of lead ions by mordenite, neither modified nor unmodified. The objective of the present research was to optimize the adsorption conditions of lead ions by mordenite. It was thought that the level of adsorption may be further increased by modification of mordenite with HDTMA. The effects of variables including initial lead concentration, adsorption time and temperature on adsorption yield were investigated by three-variable-three-level Box-Behnken Design (BBD). Emprical model correlating response to the three variables was then developed. Langmuir and Freundlich isotherm models were investigated in terms of their appropriateness and fractional-life method was used to determine kinetic parameters. The thermodynamic parameters of the process were also calculated.

## Methods

### Chemicals and reagents

Mordenite, a type of zeolite, was selected for having high ion-exchange capacity, high surface area and low Si/Al ratio. Mordenite, analytical grades of lead nitrate (Pb(NO_3_)_2_) and HDTMA were purchased from Sigma and used without further purification. Three stock lead nitrate solutions (10 mg/L, 1005 mg/L, and 2000 mg/L) were prepared and used in adsorption studies as working solutions.

### Analysis and measurements

Elemental analysis of natural mordenite was determined by Perkin-Elmer (2400 series) Elemental Analyzer. The surface area of mordenite was determined by BET analysis of Micromeritics (Gemini 2360). The concentrations of residual lead(II) ions in the supernatant solutions were determined using ICP-OES (Perkin-Elmer Optima 5300 DV) measurements.

### Design of experiments, model fitting and statistical analysis

The optimum condition for the adsorption of lead by mordenite was determined by means of BBD and RSM. RSM is a collection of statistical and mathematical techniques useful for evaluation of relationship existing between a number of controlled experimental factors and measured responses according to one or more selected criteria [15,16]. Optimization studies were carried out by studying the effect of three variables including initial lead ion concentration, adsorption temperature and time. The chosen independent variables in this study were coded according to Eq. (1):

(1) $$\chi_{i}=\frac{\chi_{i}-\chi_{0}}{\Delta \chi }$$

where x_i_ is the dimensionless coded value of the ith independent variable, x_0_ is the value of x_i_ at the center point and ∆x is the step change value. The behavior of the system is explained by the following empirical second-order polynomial model (Eq. 2):

(2) $$\begin{array}{ll} Y= & \beta_{0}+\sum_{i=1}^{k}\beta_{i}x_{i}+\sum_{i=1}^{k}\beta_{\mathrm{ii}}x_{i}^{2}\\ & +\sum_{i=1}^{k-1}\sum_{j=2}^{k}\beta_{\mathrm{ij}}x_{i}x_{j}+\epsilon \end{array}$$

where Y is the predicted response, *x*_*i*_, *x*_*j*_, …, *x*_*k*_ are the input variables, which affect the response Y, $x_{i}^{2}$, $x_{j}^{2}$,…, $x_{k}^{2}$ are the square effects, β_0_ is the intercept term, *x*_*i*_*x*_*j*_, *x*_*j*_*x*_*k*_ and *x*_*i*_*x*_*k*_ are the interaction effects, β_i_ (i = 1, 2, …, k) is the linear effect, β_ii_ (i = 1, 2, …, k) is the squared effect, β_ij_ (j = 1, 2, …, k) is the interaction effect and ϵ is a random error [17-19].

The Design-Expert 8.0 (Stat-Ease Inc., Minneapolis MN, USA) software was used for regression and graphical analysis of the experimental data to fit the equations developed and for evaluation of their statistical significance. BBD is frequently used under RSM design. The study carried out involved the employment of BBD to optimize the adsorption process due to its suitability to fit quadratic surface that usually works well for process optimization. Design of 15 experiments consisting of three replicates at the central points was employed to the second-order polynomial model. The optimum values of the selected variables were obtained by solving the regression equation at desired values of the process responses as the optimization criteria. Each of the parameters was coded at three levels: -1, 0, and +1. The range of variables was decided on the basis of pre-experimental values. The range and level of the variable in coded units for RSM studies were given in Table 1.

**Table 1:** Experimental ranges and levels of the independent variables

**Independent variables**		**Range and level**	
	-1	0	+1
Adsorption time, min (X_1_)	30	75	120
Adsorption temperature, °C (X_2_)	20	35	50
Initial lead ion concentration, mg/L (X_3_)	10	1005	2000

Each experiment was repeated three times (average values were used in optimization) and the experimental sequence was randomized in order to minimize the effects of the uncontrolled factors.

### Lead adsorption studies

Fifteen batch adsorption experiments designed by RSM were conducted with 0.50 g of mordenite (dried in an oven at 60°C for 24 h) at agitation speed of 200 rpm and the initial pH of working solution. Each experiment was carried out in Erlenmeyer flasks containing 100 ml lead(II) solution at determined temperature in an isothermal shaker. Samples were withdrawn at the end of the determined contact time and filtered through 0.25 μm filters. Filtered samples were analyzed for residual lead ion concentration. Natural mordenite particles were modified according to the reaction with 100 ml of HDTMA solution (0.05 M) at 30°C in a reactor containing 5 g mordenite particles and magnetically stirred for 3 h. At the end of the reaction, zeolite particles were separated by filtration, washed several times with distilled water and dried in an oven at 60°C for 24 h. Same adsorption procedure was applied to HDTMA-mordenite except that the conditions chosen as the optimum of unmodified one. Metal removal by mordenite was determined according to Eq. (3):

(3) $$R=\frac{P_{0}-P_{e}}{P_{0}}\times 100$$

where R is the percentage of lead adsorbed by adsorbent, P_0_ is the initial concentration of metal ion in mg/L and P_e_ is the final concentration of metal ion in mg/L.

### Adsorption isotherms

In order to diagnose the nature of adsorption (homogeneous or heterogeneous) onto the mordenite the equilibrium adsorption isotherms are required. Two theoretical isotherm models, namely Langmuir (applicable to homogeneous adsorption) and Freundlich (applicable to heterogeneous adsorption) isotherms, were used to fit the adsorption data obtained at 35°C. For Langmuir isotherm [20]:

(4) $$\frac{C_{e}}{Q}=\frac{1}{Q_{0}b}+\frac{C_{e}}{Q_{0}}$$

where C_e_ is the equilibrium concentration (mg/L), Q is the amount of metal adsorbed (mg/g), b is sorption constant (L/mg) (at 35°C) related to the energy of sorption, and Q_0_ is the maximum sorption capacity (mg/g). For Freundlich isotherm [20]:

(5) $$Q_{e}=K_{F}C_{e}^{1/n}$$

where Q_e_ is the amount of metal adsorbed at the equilibrium (mg/g), K_F_ ((mg/g)(L/mg)^1/n^) and n (dimensionless) are Freundlich constants related to the adsorption capacity and adsorption intensity, respectively. The regression analysis and calculation of constants of Eq. (4) and (5) were achieved by using the solver add-in function of MS Excel.

### Adsorption kinetics

The adsorption data obtained with 1005 mg/L initial lead ion concentration were then fitted to the fractional-life method [21]:

(6) $$t_{F}=\frac{F^{1-n}-1}{k\left(n-1\right)}\;C_{A0}^{1-n}$$

where F is the fractional value (C_A_/C_A0_) in time t_F_; n the reaction order; k reaction rate constant; and C_A_ the concentration of reactant A (mg/L) at time t (min). Kinetic parameters of adsorption were determined by plotting log t_F_ versus log C_A0_ and using MS Excel.

### Adsorption thermodynamics

Since temperature is an important parameter effecting both adsorption capacity of mordenite and transport/kinetic process of lead adsorption, the thermodynamic parameters of the process such as enthalpy (∆H˚), entropy (∆S˚), and free energy (∆G˚) of lead adsorption on mordenite were calculated by using following thermodynamic relations [19,22]:

(7) $$\Delta G^{0}=-\mathrm{RT}\;{\mathrm{In}\;K}_{c}^{0}$$

(8) $$\Delta G^{{}^{\circ}}=\Delta H^{{}^{\circ}}-T\Delta S^{{}^{\circ}}$$

Combining these to relations:

(9) $$\mathrm{In}\;K_{c}^{0}=\frac{\Delta S^{0}-\Delta H^{0}}{R}*\frac{1}{T}$$

where R is universal gas constant (8.314 Jmol^-1^ K^-1^), T is the temperature (K) and K_c_ is the equilibrium constant at that temperature calculated by the equation:

(10) $$K_{c}=\frac{C_{a}}{C_{e}}$$

where C_a_ is the concentration of the adsorbed material (mg/L), and C_e_ is the concentration of remaining material in solution (mg/L).

## Results

### Properties of natural and modified mordenite

Natural mordenite has a purity of 99.8 percent with impurity of magnesium. According to the chemical composition of the zeolite, the chemical formula was determined as K_2.87_Ca_1.43_Na_1.27_Al_7.98_Si_40_O_88_.25H_2_O. This result showed that the major components of mordenite were silica and alumina and the ratio of Si/Al was 5.215. The surface area of the mordenite was determined as 53 m^2^/g with BET analysis. HDTMA-modification was corrected with the elemental analysis [23]. The ratio of C/N for HDTMA-mordenite showed that 4.15 percent of modifying agent was transferred onto the surface of the zeolite.

### Regression model and optimization

The coded and actual values of the test variables and lead removal percentage were summarized in Table 2. Predicted values for yield by using quadratic model and reduced cubic model were summarized in Tables 3 and 4, respectively. The statistical significance of the quadratic and reduced cubic models was evaluated by analysis of variance (ANOVA) as presented in Table 5 and 6, respectively.

**Table 2:** Experimental design matrix based on Box-Behnken and results

**Run no**	**Independent variables**						**Observed values**
	**Coded values**			**Real values**			
1	-1	0030	-1	30	35	10	55.800
2	0	+1	-1	75	50	10	83.500
3	+1	0	-1	120	35	10	71.400
4	0	+1	+1	75	50	2000	49.350
5	0	0	0	75	35	1005	48.498
6	-1	+1	0	30	50	1005	47.190
7	0	0	0	75	35	1005	48.373
8	+1	0	+1	120	35	2000	48.900
9	+1	-1	0	120	20	1005	44.530
10	0	0	0	75	35	1005	48.385
11	0	-1	-1	75	20	10	45.200
12	+1	+1	0	120	50	1005	49.930
13	-1	-1	0	30	20	1005	42.915
14	-1	0	+1	30	35	2000	47.700
15	0	-1	+1	75	20	2000	44.800

**Table 3:** Observed and predicted values for the quadratic model

**Run no**	**Observed values**	**Predicted value**	**Residual**
1	55.800	57.850	-2.05
2	83.500	78.860	4.64
3	71.400	70.340	1.06
4	49.350	45.700	3.65
5	48.498	48.420	0.079
6	47.190	49.780	-2.59
7	48.373	48.420	-0.046
8	48.900	46.850	2.05
9	44.530	41.940	2.59
10	48.385	48.420	-0.034
11	45.200	48.850	-3.65
12	49.930	55.630	-5.70
13	42.915	37.210	5.70
14	47.700	48.760	-1.06
15	44.800	49.440	-4.64

**Table 4:** Observed and predicted values for the reduced cubic model

**Run no**	**Observed values**	**Predicted value**	**Residual**
1	55.800	55.800	0.000
2	83.500	83.500	0.000
3	71.400	71.400	0.000
4	49.350	49.350	0.000
5	48.498	48.420	0.078
6	47.190	47.190	0.000
7	48.373	48.420	-0.047
8	48.900	48.900	0.000
9	44.530	44.530	0.000
10	48.385	48.420	-0.035
11	45.200	45.200	0.000
12	49.930	49.930	0.000
13	42.915	42.920	0.005
14	47.700	47.700	0.000
15	44.800	44.800	0.000

**Table 5:** Analysis of variance (ANOVA) for the quadratic model

**Source of variations**	**Degrees of freedom**	**Sum of squares**	**Mean square**	**F-value**	**Probability (p)**
Regression	9	1560.78	173.42	5.46	0.0381
Main effects	3	931.37	931.37	29.31	0.2419
Square effects	3	279.68	279.68	8.80	0.7422
Interaction effects	3	336.93	336.93	10.60	0.9244
Residual	5	158.89	31.78		
Total	14	1719.67			

R^2^; 0.9076, adjusted R^2^; 0.7413, predicted R^2^; -0.4783, lack of fit; 11134.81, CV; 10.89.

**Table 6:** Analysis of variance (ANOVA) for the reduced cubic model

**Source of variations**	**Degrees of freedom**	**Sum of squares**	**Mean square**	**F-value**	**Probability (p)**
Regression	12	1719.66	143.30	30129.29	<0.0001
Main effects	3	828.02	828.02	174087.1	<0.0001
Square effects	3	279.68	279.68	58801.17	0.0012
Interaction effects	3	336.93	336.93	70836.5	0.0147
Added terms	3	158.88	158.88	33404.44	0.0024
Total	14	1719.67			

R^2^; 1.000, adjusted R^2^; 1.000, CV; 0.13.

According to the statistical analysis, the empirical relationship in coded units obtained by RSM was as follows:

(11) $$\begin{array}{ll} Y= & 48.42+4.20x_{1}+10.71x_{2}-8.64x_{3}\\ & +0.28x_{1}x_{2}-3.60x_{1}x_{3}-8.44x_{2}x_{3}-1.02x_{1}^{2}-1.26x_{2}^{2}\\ & +8.55x_{3}^{2}-8.29x_{1}^{2}x_{2}+0.99x_{1}^{2}x_{3}-3.11x_{1}x_{2}^{2} \end{array}$$

where Y is lead ion removal (response) in percentage, *x*_1_, *x*_2_ and *x*_3_ are the coded values of variables; adsorption time in min (*x*_1_), adsorption temperature in °C (*x*_2_), and initial lead ion concentration in mg/L (*x*_3_).

**Figure 1:**
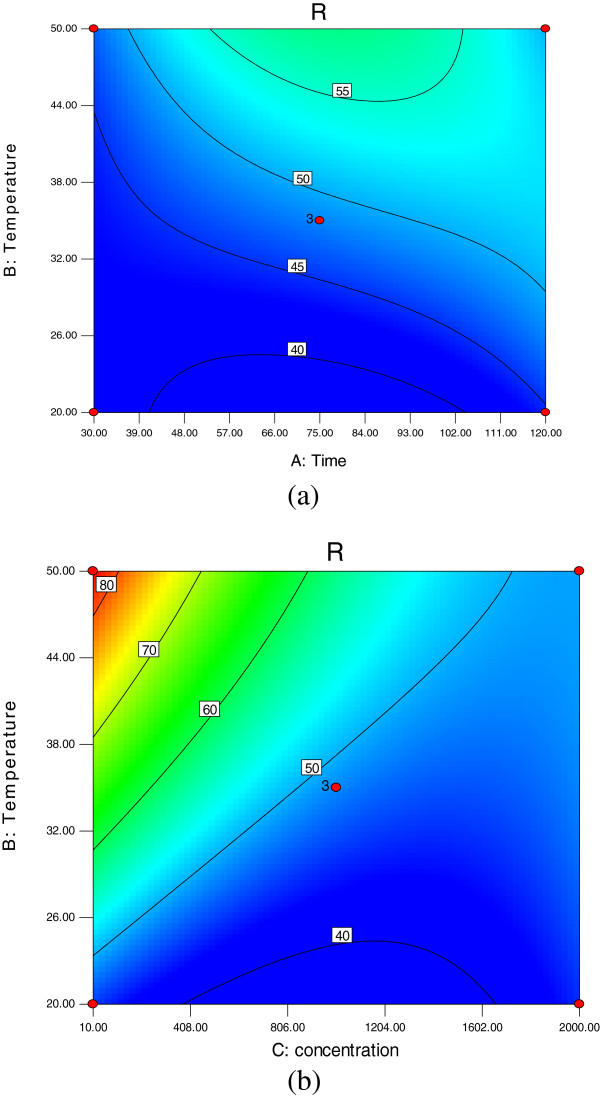
Contour plots for the effect of (a) adsorption temperature (°C) and adsorption time (min); (b) adsorption temperature (°C) and initial lead ion concentration (mg/L) on the lead removal (black-and-white form).

The results of regression analysis on reduced cubic model are given in Table 7. The significant of each coefficient was determined by F-values and p-values (Table 7). The larger the magnitude of the F-values and the smaller p-values, the more significant is the corresponding coefficients. Values of “prob > F” less than 0.0500 also indicated high significant regression at 95 percent confidence level. According to the F- and p-values, adsorption temperature and initial ion concentration were found more effective on the adsorption process. Figure 1 shows the simultaneous effects of adsorption temperature (a) and initial lead ion concentration (b) on lead removal efficiency.

**Table 7:** Regression analysis for the reduced cubic model

**Model term**	**Coefficient estimate**	**Standart error**	**F-Value**	**p-Value**
Intercept	+48.42	0.04	30129.29	<0.0001
*x*_1_	+4.20	0.034	14834.96	<0.0001
*x*_2_	+10.71	0.034	96509.35	<0.0001
*x*_3_	-8.64	0.034	62742.79	<0.0001
*x*_1_*x*_2_	+0.28	0.034	66.52	0.0147
*x*_1_*x*_3_	-3.60	0.034	10899.15	<0.0001
*x*_2_*x*_3_	-8.44	0.034	59870.83	<0.0001
$x_{1}^{2}$	-1.02	0.036	807.59	0.0012
$x_{2}^{2}$	-1.26	0.036	1227.48	0.0008
$x_{3}^{2}$	+8.55	0.036	56766.10	<0.0001
$x_{1}^{2}x_{2}$	-8.29	0.049	28924.08	<0.0001
$x_{1}^{2}x_{3}$	+0.99	0.049	410.05	0.0024
$x_{1}x_{2}^{2}$	-3.11	0.049	4070.31	0.0002

In the numerical optimization desirability function was used. In order to determine the usability of mordenite in low and high concentrated lead solutions, time and temperature were set within range, and level of lead removal was set at maximum in each criteria (Table 8). Initial lead ion concentration was set as different targets for maximum desirability. By seeking from several starting points for each criteria, the best local maximums for each were obtained and summarized in Table 8.

**Table 8:** Numerical optimization of the model obtained by desirability function

**Criteria**		**Solution**	**Desirability**
1	Adsorption time: in range	89.47	1.000
	Adsorption temperature: in range	49.68	
	Initial lead concentration: target = 10	10.00	
	Lead removal: maximize	83.7369	
2	Adsorption time: in range	85.29	0.968
	Adsorption temperature: in range	50.00	
	Initial lead concentration: target = 100	100.00	
	Lead removal: maximize	81.0066	
3	Adsorption time: in range	81.83	0.602
	Adsorption temperature: in range	50.00	
	Initial lead concentration: target = 1000	799.10	
	Lead removal: maximize	61.8751	
4	Adsorption time: in range	82.66	0.425
	Adsorption temperature: in range	50.00	
	Initial lead concentration: target = 2000	828.11	
	Lead removal: maximize	61.2467	

### Characterization of adsorption

In the present study, Langmuir and Freundlich isotherm models [20] were applied to the experimental data to determine the type of adsorption (homogeneous, heterogeneous). The adsorption constants of each isotherm were summarized in Table 9. According to those, the value of R^2^ so lower than 1, demonstrating that Langmuir isotherm is not applicable for describing the adsorption. The experimental data fitted well to Freundlich Model (R^2^ = 0.993) which shows that mordenite has a form of surface heterogeneity [20].

**Table 9:** Isotherm model constants for the adsorption of lead on mordenite (T: 35°C, sorbent dosage: 0.5 g, initial lead concentration: 10–2000 mg/L)

**Langmuir model**			**Freundlich model**		
Q_0_ (mg/g)	b (L/mg)	R^2^	1/n	K_F_	R^2^
4.387	0.0006	0.571	0.876	0.0041	0.993

**Figure 2:**
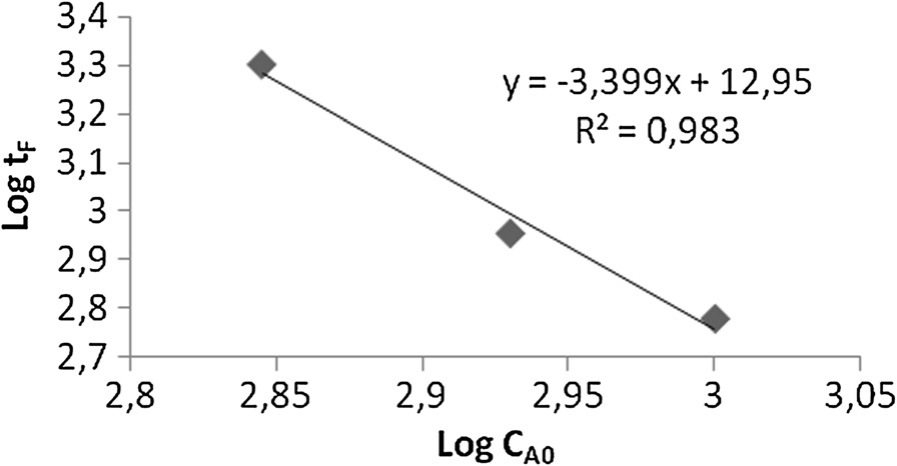
Lead adsorption reaction rate at 0°C (initial lead concentration 1005 mg/L) (t_F_ (min); c_A0_ (mg/L).

The adsorption kinetics was determined by fractional-life method chosing the fractional value of 0.8 [21]. The reaction rate was 4.39 at 0°C for 1005 mg/L of initial lead concentration (Figure 2) and the rate constant was calculated as 3.75.10^-14^ min^-1^(mg/L)^-3.39^.

The thermodymanics of adsorption of lead on mordenite was studied at temperatures of 20, 35, and 50°C. The change in Gibbs free energy (∆G˚), enthalpy (∆H˚), and entropy (∆S˚) values were obtained by using Eq. (9) and the results were summarized in Table 10.

**Table 10:** Thermodynamical constants of lead adsorption at different temperatures

**T (°C )**	**Kc**^**0**^	**ΔG**^**0**^** (kJ/mol)**	**ΔS**^**0**^	**ΔH**^**0**^
20	0.791	571.44	15.91	-4737
35	1.683	-1333.70		
50	3.55	-3403.87		

## Discussion

In order to develop an equation describing the relation between the adsorption yield and three adsorption variables shown in Table 2, a BBD was conducted. Quadratic model and reduced cubic model were developed to correlate the variables to the response. Predicted values for yield by using reduced cubic model (Table 4) were closer to observed values than those by using quadratic model (Table 3). In quadratic model (Table 5), F-value of the model 5.46 implied that the model was statistically significant. There was only a 3.81 percent chance that a model F-value this large could occur due to noise. The fit of the model was checked by the determination coefficient (R^2^). In this case, the value of the determination coefficient (R^2^ = 0.9076) indicated that 9.24 percent of the total variable was not explained by the model. The value of adjusted determination coefficient (adjusted R^2^ = 0.7413) was low and it was not in reasonable agreement with the adjusted R^2^. Negative predicted R^2^ implied that the overall mean was a better predictor of the response than the current model. Significant lacks of fit and high value of the coefficient of variation were found. Probability values (greater than 0.1000) indicated that some of the model terms are not significant. But, reduced cubic model (Table 6) produced the closest predicted values, predicted R^2^ and adjusted R^2^ equal to 1, no lack of fit and low coefficient of variance. The model F-value implied that the model was significant, and there was only a 0.01 percent chance occurs due to noise. Thus, as a result of the statistical analysis, reduced cubic model was found satisfactory for describing the process and useful for developing empirical relation.

Lead removal showed to be very sensitive to changes in the temperature both in dilute and in concentrated solutions. The removal capacity of mordenite was sharply increased when the adsorption temperature increased from 20 to 50°C in dilute solutions; as it was also reported by Wang [14] (from 20 to 40°C; in a solution of 40 mg lead/L). No comparison can be made with the research by Dai *et al*. [13] since the temperature set constant at 25°C. For moderately concentrated solutions (1005 mg/L) this increase in yield was only 5 percent. The increase in yield due to increase in adsorption temperature in diluted solutions was more dominated than one in concentrated solutions.

Initial lead ion concentration was another parameter that has high effect on the response. As can be seen on Figure 1, the concentration of the aqueous solution increases the removal of lead decreases. About 84 percent yield was obtained in diluted solutions (10 mg/L). When the initial lead concentration increased to 100 mg/L, the yield decreased to 81 percent. Dai *et al*. [13] also observed that increase in initial lead concentration from 3 to 200 mg/L decreased the lead removal down to 70 percent. Also (Figure 1), the results of the study showed that this yield could also be achieved up to 500 mg/L of initial lead concentrations.

Adsorption time has little effect on lead removal. It was found that nearly 80–90 minute was enough to obtain highest yield in both dilute and concentrated solutions. The results obtained were in agreement with the work done by Dai [13] which reported that optimum time required to reach the equilibrium was 100 min. Also Wang [14] reported that adsorption time has little effect on yield and that the adsorption required 90 min.

Optimum conditions for the adsorption process were searched by numerical optimization section of the software by choosing different targets for initial lead ion concentration. As shown in Table 8, for dilute solutions (initial lead ion concentration up to 10 mg/L), the best local maximum was found to be at adsorption time of 85–90 min, adsorption temperature of 50°C, lead removal of nearly 84 percent, and desirability of 1.000. High desirability shows that the estimated function may represent the experimental model and desired conditions. As the concentration increases, desirability and lead removal percentage were found to be decreasing (Table 8). Finally, 97 percent lead adsorption was achieved by using HDTMA-mordenite at the same optimum conditions of unmodified-mordenite; i.e. initial lead ion concentrations: 10–100 mg/L. It can be concluded that, modification of mordenite or adjustment of adsorption medium (lowering up to pH 3) [13] produce nearly the same adsorption yield. But lowering the pH of the medium was useful for solutions containing 3 mg lead /L [13], whereas modification of mordenite was applicable up to 100 mg/L of lead solutions.

Fitting of adsorption data showed that (Table 9), Langmuir isotherm, thus monolayer adsorption, solely, was not suitable for the process. Equilibrium adsorption data were best represented by the Freundlich isotherm. Heterogeneous adsorption of lead on mordenite was also stated in the literature before [14]. The obtained value of (1/n) (0.1 < 1/n < 1) demonstrated that favourable nature of both lead and the heterogeneity of the mordenite sites. The 1/n value of the present study was higher than those obtained (0.537 at 30°C; 0.555 at 40°C) [14] showing that higher adsorption intensity of the mordenite used. Negative Gibbs free energy (Table 10) indicated the spontaneous nature of adsorption at those temperatures. These results were well-matched with literature [14]. Also, negative ∆H˚ values showed the adsorption of lead ions was an exothermic process. A negative enthalpy values were also reported for the adsorption of lead ions onto wollastonite, bentonite and mordenite [14]. A positive ∆S˚ value corresponded to an increase in both the randomness at the solid-solution interface and the degree of freedom of the adsorbed species. Adsorption reaction rate was found 4.4 at 0°C (Figure 2). In a different study, lead adsorption data (at 20-40°C) onto a local mordenite were fitted well to several equations; pseudo-second order, parabolic diffusion and Elovich equations [14]. It seemed that the steps of adsorbate transport from the solution to the surface of mordenite; such as film diffusion, pore diffusion, surface diffusion and adsorption are strongly affected on temperature.

## Conclusions

A Box-Behnken Design was conducted to study the effects of three adsorption variables, namely adsorption time, temperature and initial lead ion concentration on the adsorption yield of lead. Quadratic model and reduced cubic model were developed to correlate the variables to the response. Through the analysis of response surfaces, adsorption temperature and initial lead ion concentration were found to have significant effects on adsorption yield, whereas initial ion concentration showed that most significant. Process optimization was carried out and the experimental values were found to agree satisfactorily with the predicted values. Mordenite was shown to be a promising adsorbent for removal of lead from aqueous solutions. Further increase in the adsorption yield obtained at the optimum conditions was achieved with HDTMA-modification. Adsorption isotherm, adsorption kinetic and adsorption thermodynamics were studied. Equilibrium adsorption data were best represented by the Freundlich isotherm model. Thermodynamic calculations indicated that the adsorption was exothermic and spontaneous process.

## Competing interests

The authors declared that they have no competing interests.

## Authors’ contributions

HT carried out part of the experimental studies. TK carried out part of the experiments and participated in the design of the study. SYY performed the statistical analysis, conceived of the study, and participated in its design and coordination and helped to prepare the manuscript. All authors read and approved the final manuscript.

## References

[B1] Förstnet U Wittmann GTW Metal pollution in the aquatic environment 1979 Berlin: Springer Verlag

[B2] Bahadir T Bakan GL Aitas H The investigation of lead removal by biosorption: an application at storage battery industry wastewaters Enzyme Microb Technol 2007 41 98 102 10.1016/j.enzmictec.2006.12.007

[B3] Liu YG Fan T Zeng G Li X Tong Q Ye F Zhou M Xu W Huang Y Removal of cadmium and zinc ions from aqueous solution by living *Aspergillus niger* Trans Nonferrous Met Soc China 2006 16 681 686 10.1016/S1003-6326(06)60121-0

[B4] Wang J Chen C Biosorption of heavy metals by *Saccharomyces cerevisiae*: a review Biotechnol Adv 2006 24 427 451 10.1016/j.biotechadv.2006.03.001 16737792

[B5] Chen J Wang L Zhou S Determination of lead biosorption properties by experimental and modelling simulation study Chem Eng J 2007 131 209 215 10.1016/j.cej.2006.11.012

[B6] Zhou P Huang JC Li AWF Wie S Heavy metal removal from wastewater in a fluidized bed reactor Water Res 1999 33 1918 1924 10.1016/S0043-1354(98)00376-5

[B7] Mousavi HZ Hosseynifar A Jahed V Dehghani SAM Removal of lead from aqueous solution using waste tire rubber ash as an adsorbent Braz J Chem Eng 2010 27 79 87

[B8] Crini G Non-conventional low-cost adsorbents for dye removal: a review Bioresour Technol 2006 97 1061 1085 10.1016/j.biortech.2005.05.001 15993052

[B9] Wang S Peng Y Natural zeolites as effective adsorbents in water and wastewater treatment: review Chem Eng J 2010 156 11 24 10.1016/j.cej.2009.10.029

[B10] Godelitsas A Armbruster T HEU-type zeolites modified by transition elements and lead Micropor Mesopor Mater 2003 61 3 24 10.1016/S1387-1811(03)00352-4

[B11] Wang XS Hu HQ Sun C Removal of Cu (II) ions from aqueous solutions using Na-mordenite Sep Sci Technol 2007 42 1215 1230 10.1080/01496390701241956

[B12] Wang XS Huang J Hu HQ Qin Y Determination of kinetic and equilibrium parameters of the batch adsorption of Ni (II) from aqueous solutions by Na-mordenite J Hazard Mater 2007 142 468 476 10.1016/j.jhazmat.2006.08.047 17010513

[B13] Dai S Kato Y Guang D Amal R Removal of lead (II) by mordenites Dev Chem Eng Mineral Process 1998 6 171 184

[B14] Wang XS He L Hu HQ Wang J Effect of temperature on the Pb(II) removal from single aqueous solutions by a locally natural mordenite: equilibrium and kinetic modelling Separ Sci Technol 2008 43 908 922 10.1080/01496390701870697

[B15] Bas D Boyaci İH Modelling and optimisation I. Usability of response surface methodology J Food Eng 2007 78 836 845 10.1016/j.jfoodeng.2005.11.024

[B16] Preetha B Viruthagiri T Application of response surface methodology for the biosorption of cupper using *Rhisopus arrhizus* J Hazard Mater 2007 143 506 510 10.1016/j.jhazmat.2006.09.077 17084526

[B17] Goksungur Y Uren S Guvenc U Biosorption of cadmium and lead ions by ethanol treated waste baker’s yeast biomass Bioresource Technol 2005 96 103 109 10.1016/j.biortech.2003.04.002 15364087

[B18] Aksu Z Gonen F Binary biosorption of phenol and chromium (VI) onto immobilized activated sludge in a packed bed: prediction of kinetic parameters and breakthrough curves Sep Purif Technol 2006 49 205 216 10.1016/j.seppur.2005.09.014

[B19] Aksu Z Determination of the equilibrium, kinetic and thermodynamic parameters of the batch biosorption of nickel (II) ions onto *Chorella vulgaris* Process Biochem 2002 38 89 99 10.1016/S0032-9592(02)00051-1

[B20] Benefield L Judkins J Weand B Process chemistry for water and wastewater treatment 1982 New York: Prentice Hall

[B21] Levenspiel O Chemical reaction engineering 1999 United States of America: John Wiley and Sons

[B22] Dakiky M Khamıs M Manassra A Mer’eb M Selective adsorption of chromium (VI) in industrial wastewater using low-cost abundantly available adsorbents Advances Environ Res 2002 6 533 540 10.1016/S1093-0191(01)00079-X

[B23] Gok O Ozcan AS Ozcan A Adsorption behaviour of a textile dye of Reactive Blue 19 from aqueous solutions onto modified bentonite Appl Surf Sci 2010 256 5439 5443 10.1016/j.apsusc.2009.12.134

